# Cryopreservation of artificial gut microbiota produced with *in vitro* fermentation technology

**DOI:** 10.1111/1751-7915.12844

**Published:** 2017-10-04

**Authors:** Lea Bircher, Clarissa Schwab, Annelies Geirnaert, Christophe Lacroix

**Affiliations:** ^1^ Laboratory of Food Biotechnology Institute of Food, Nutrition and Health ETH Zürich Schmelzbergstrasse 7 8092 Zürich Switzerland

## Abstract

Interest in faecal microbiota transplantation (FMT) has increased as therapy for intestinal diseases, but safety issues limit its widespread use. Intestinal fermentation technology (IFT) can produce controlled, diverse and metabolically active ‘artificial’ colonic microbiota as potential alternative to common FMT. However, suitable processing technology to store this artificial microbiota is lacking. In this study, we evaluated the impact of the two cryoprotectives, glycerol (15% v/v) and inulin (5% w/v) alone and in combination, in preserving short‐chain fatty acid formation and recovery of major butyrate‐producing bacteria in three artificial microbiota during cryopreservation for 3 months at −80°C. After 24 h anaerobic fermentation of the preserved microbiota, butyrate and propionate production were maintained when glycerol was used as cryoprotectant, while acetate and butyrate were formed more rapidly with glycerol in combination with inulin. Glycerol supported cryopreservation of the *Roseburia* spp./*Eubacterium rectale* group, while inulin improved the recovery of *Faecalibacterium* *prausnitzii*. *Eubacterium hallii* growth was affected minimally by cryopreservation. Our data indicate that butyrate producers, which are key organisms for gut health, can be well preserved with glycerol and inulin during frozen storage. This is of high importance if artificially produced colonic microbiota is considered for therapeutic purposes.

## Introduction

Pathogenesis of several gastrointestinal diseases has been linked to functional alterations and compositional imbalances of the intestinal microbiota, also referred as dysbiosis. For example, decreased microbial diversity has been associated with recurrent *Clostridium difficile* infection (RCDI) (Milani *et al*., 2016). Changes in the abundance of the butyrate‐producing bacterial community along with reduced butyrate formation were observed in inflammatory bowel disease (IBD) (Sokol *et al*., 2009; Kumari *et al*., 2013; Fuentes *et al*., 2017). To restore the microbial balance, transfer of faecal microbiota from a healthy donor (FMT) to diseased patients has been suggested as a therapeutic strategy. The success of FMT in treating RCDI has been demonstrated in several studies, with cure rates exceeding 90% (van Nood *et al*., 2013; Kelly *et al*., 2016; Lee *et al*., 2016). Use of FMT as a therapy for other gastrointestinal disorders such as IBD has been proposed (Vermeire *et al*., 2016). To date, safety concerns and acceptability are main constraints of therapeutic uses of FMT. Fresh faecal matter is preferably obtained from relatives of the patients immediately before transplantation. A careful donor screening regarding faecal microbiota composition, pathogen status and undesirable antigens and ‘phenotypes’ must be performed preventively (Petrof and Khoruts, 2014; Alang and Kelly, 2015). Despite the increasing demand for FMT, rigorous exclusion criteria for donors strongly limit the widespread availability of suitable faecal material (Konig *et al*., 2017). The approach of transplanting ‘artificially’ produced microbiota, which has been extensively characterized, might alleviate these limitations. Continuous *in vitro* intestinal fermentation technology (IFT) with immobilized faecal microbiota, mimicking both the planktonic and sessile growth, can be used to produce controlled and stable ‘artificial’ colonic microbiota at high cell density and at large quantity (Cinquin *et al*., 2006; Payne *et al*., 2012; Zihler Berner *et al*., 2013; Fehlbaum *et al*., 2015; Lacroix *et al*., 2015). Nevertheless, processing for long‐term preservation is required to guarantee availability of artificial faecal microbiota for transplantation.

Cryopreservation at temperatures ranging from −80°C in electrical freezers to −196°C in liquid nitrogen is a widely used method for storing bacteria (Prakash *et al*., 2013). However, ice formation during freezing can cause lethal damage to bacterial cells; thus, cryoprotectants must be added to prevent cryoinjuries. The positive effects of different protective matrices composed out of polysaccharides, amino acids, peptides or more complex compounds on microbial cell physiology during freezing, storage and thawing have been studied for pure cultures and are well approved (Hubalek, 2003). Non‐penetrating cryoprotectants, such as many saccharides, reduce ice formation within cells by osmosis‐derived dehydration before freezing. Cryoprotective sugars can also bind to the cell surface and inhibit extracellular ice crystal formation. Penetrating cryoprotectants, such as glycerol and dimethyl sulfoxide (DMSO), change fluid properties and increase membrane glass‐phase transition temperature, which results in reduced intracellular ice formation (Hubalek, 2003; Fowler and Toner, 2005). Nevertheless, DMSO is not recommended for *in vivo* administrations as it was found to be toxic to cells at low concentrations of 2–4% (Galvao *et al*., 2014), whereas glycerol is a common additive for frozen faecal samples also in stool banks (Aguirre *et al*., 2015). The clinical efficacy of cryopreserved faecal slurries for resolving RCDI has been investigated before. It was shown that preserved slurries with or without glycerol exhibited remission rates similar to fresh FMT. Furthermore, only minor viability drops in the tested cultivable anaerobes over 6 months of frozen storage were reported (van Nood *et al*., 2013; Youngster *et al*., 2014; Costello *et al*., 2015; Satokari *et al*., 2015). However, artificially produced microbiota may lack the protective effect of a matrix naturally present in stool. Therefore, composition of the protective matrix for cryopreservation of artificially produced microbiota should be investigated.

In this work, we observed the effect of two cryoprotective agents on maintaining metabolic activity of artificial colonic microbiota produced with IFT. Glycerol and inulin were chosen as penetrating and non‐penetrating cryoprotective agents respectively as combining compounds with different mechanisms can result in additive or synergic protective effects (Hubalek, 2003). Glycerol was selected due to its low toxicity to microbial and human cells and its widespread use for frozen stool samples intended for FMT (Hamilton *et al*., 2012). Inulin acts extracellularly by stabilizing membranes via interplay with membrane lipids and providing mechanical protection from enhanced surface pressure (Demel *et al*., 1998) and might substitute for a protective matrix.

Moreover, we also investigated the impact of cryopreservation on the re‐establishment of the major butyrate producers *Faecalibacterium prausnitzii* (*Clostridium* cluster IV), *Eubacterium hallii* and the *Roseburia* spp./*Eubacterium rectale* (*Clostridium* cluster XIVa). Butyrate is an important short‐chain fatty acid (SCFA) that provides several benefits to the host (Tan *et al*., 2014). Butyrate producers of the *Clostridium* clusters IV and XIVa have been associated with a ‘sustained response’ to FMT in IBD and could therefore be crucial in restoring the metabolic balance of a disturbed intestinal microbiota (Fuentes *et al*., 2017). In this study, three different colonic microbiota, originating from two *in vitro* gut fermentations inoculated with immobilized faecal microbiota of two healthy adult donors, were preserved in buffers containing either glycerol or inulin or a combination thereof. Metabolite formation, as marker of metabolic activity of the processed artificial microbiota, and re‐establishment of selected butyrate producers were determined before and after storage at −80°C to identify the specific effect of cryopreservation on SCFA production and on growth of selected butyrate producers as well as to observe the protective potential of the added cryoprotectants.

## Results

### Bacterial composition of artificial gut microbiota

**Figure 1:**
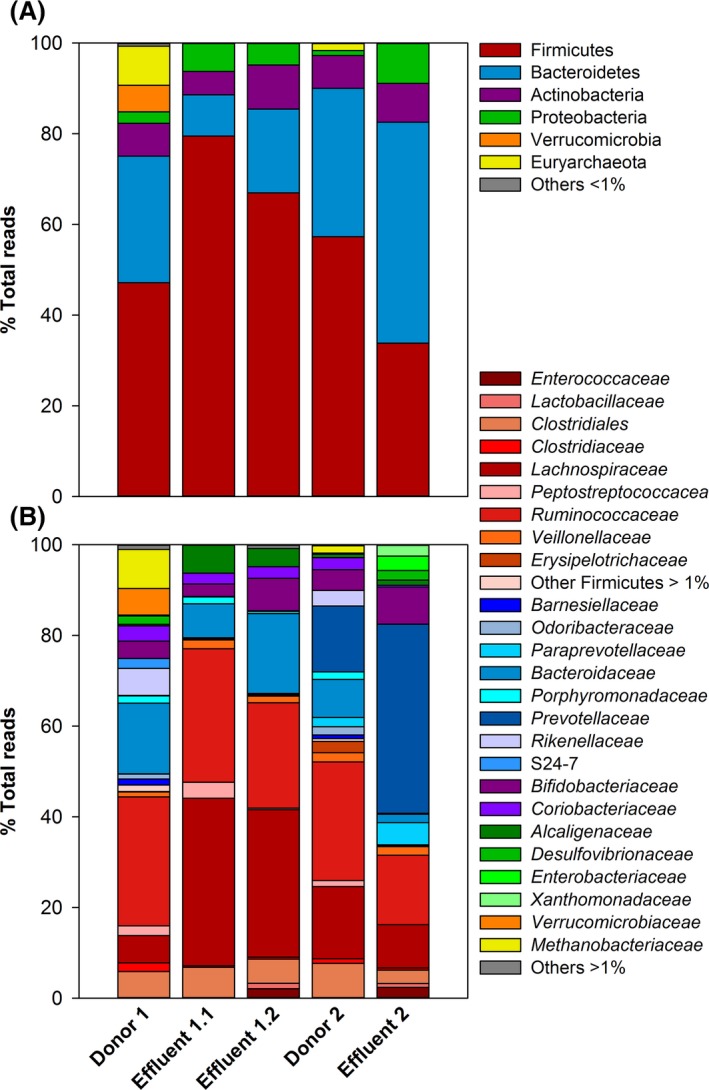
Microbial composition of donor faeces and fermentation effluents. Relative abundance of microbial phyla (A) and families (B) in the fermentation effluents and corresponding donors was analysed by V4 region 16S rRNA gene sequencing.

Two continuous colonic fermentation systems (F1 and F2) consisting of single reactors mimicking conditions of the proximal colon were inoculated with immobilized faecal microbiota from two healthy adult male donors and used to produce artificial gut microbiota. Two freezing trials were carried out using two effluents of F1 obtained with standard fermentation conditions (effluent 1.1) after reaching steady‐state operation; or after pH stress application (effluent 1.2), which shifted microbiota composition and metabolic activity. One freezing trial was conducted with effluents of F2 obtained after reaching steady‐state operation (effluent 2). Microbial composition of both fermenter effluents and donor faeces was determined by sequencing the V4 region of the 16S rRNA gene amplicons (Fig. 1). In addition, quantitative real‐time PCR (qPCR) was performed to investigate the relative abundance of butyrate‐producing bacteria, *F. prausnitzii*,* E. hallii* and the *Roseburia* spp./*E. rectale* group in the fermentation effluents (Table 1).

**Table 1:** Relative abundance of *Roseburia spp*./*E. rectale* group, *F. prausnitzii* and *E. hallii* in fermentation effluents

Targeted butyrate‐producing bacteria	Relative abundance^a^		
	Effluent 1.1	Effluent 1.2	Effluent 2
*Roseburia* spp./*E. rectale*	0.7	0.1	0.0
*F. prausnitzii*	11.0	5.6	3.1
*E. hallii*	0.0	0.0	0.0

*But* genes of *Roseburia* spp./*E.rectale* and *F. prausnitzii* and 16S rRNA of *E. hallii* were targeted by qPCR. Relative abundance was calculated relative to total 16S rRNA gene copies. Results for *but* genes were multiplied by five to account for multiple 16S rRNA gene copies.

a Percentage of total bacteria numbers from single measurements.

The microbiota in effluent 1.1 was mainly Firmicutes (79.5% of the reads). Bacteroidetes was the second most abundant phylum, accounting for 9.1% of the reads. *Ruminococcaceae* (29.5%) and *Lachnospiraceae* (36.9%) were the most prevalent families. In effluent 1.2, the abundance of Firmicutes was lower, at 67.0% of the total reads, and was represented by the families *Ruminococcaceae* (23.2%) and *Lachnospiraceae* (32.5%). Bacteroidetes were higher in effluent 1.2 (18.5%) compared to effluent 1.1. In contrast to F1, effluent from F2 was predominated by Bacteroidetes (48.7%) and contained 33.9% Firmicutes. At the family level, *Prevotellaceae* (41.7%) were predominant, followed by *Ruminococcaceae* and *Lachnospiraceae*, which represented 15.3 and 9.5% of the reads respectively (Table S1 and S2).

*F. prausnitzii* represented 11.0%, 5.6% and 3.1% of the bacterial community in effluent 1.1, 1.2 and 2 respectively (Table 1). Relative abundance of the *Roseburia* spp./*E. rectale* group ranged from 0.8% to below 0.1%, while relative abundance of *E. hallii* was between 0.002% and 0.005%.

Effluents 1.1 and 1.2 exhibited a lower diversity (Shannon index of 3.2 and 3.1 respectively) compared to the corresponding faecal sample 1 (Shannon index 4.2). Transferring faecal microbiota from donor sample to reactor decreased the Bacteroidetes:Firmicutes ratio, from 0.6 in the faecal inoculum to 0.1 in effluent 1.1. In F2, the Bacteroidetes:Firmicutes ratio shifted from 0.6 in faeces to 1.4 in effluent 2, mainly due to a decrease in *Ruminococcaceae* and an increase in *Prevotellaceae*. Overall diversity decreased in effluent 2 compared to faeces, with respective Shannon indices of 2.7 and 4.2.

### Metabolic profile of artificial gut microbiota

**Figure 2:**
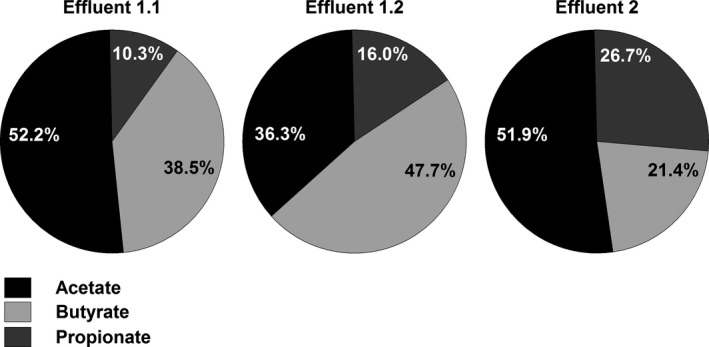
Percentage of main metabolites in fermentation effluent samples. Ratios of major metabolites acetate, propionate and butyrate were calculated from the concentrations measured by HPLC‐RI giving an acetate:propionate:butyrate ratio of 1:0.2:0.8 for effluent 1.1, 1:0.4:1.3 for effluent 1.2 and 1:0.5:0.4 for effluent 2.

The metabolite profiles in fermentation effluents were analysed by high‐performance liquid chromatography with refractive index detection (HPLC‐RI) (Fig. 2). Concentrations of main SCFAs, acetate, butyrate and propionate, differed between effluents (Table S3). Intermediate metabolites, lactate and formate, were present at low concentrations (≤1.0 mM) or were not detected. SCFAs of effluent 1.1 were dominated by acetate (62.3 mM) and were characterized by a high concentration of butyrate (46.8 mM) and a low level of propionate (12.5 mM). The predominant SCFAs of effluent 1.2 were butyrate (56.6 mM), acetate (43.1 mM) and propionate (19.0 mM), resulting in acetate:propionate:butyrate ratios of 1:0.2:0.8 for effluent 1.1 and 1:0.4:1.3 for effluent 1.2. Effluent 2 contained 36.6 mM propionate, 29.3 mM butyrate and a high concentration of acetate (71.1 mM), giving an acetate:propionate:butyrate ratio of 1:0.5:0.4.

### SCFA formation of fermentation effluents prior to and postfreezing

**Figure 3:**
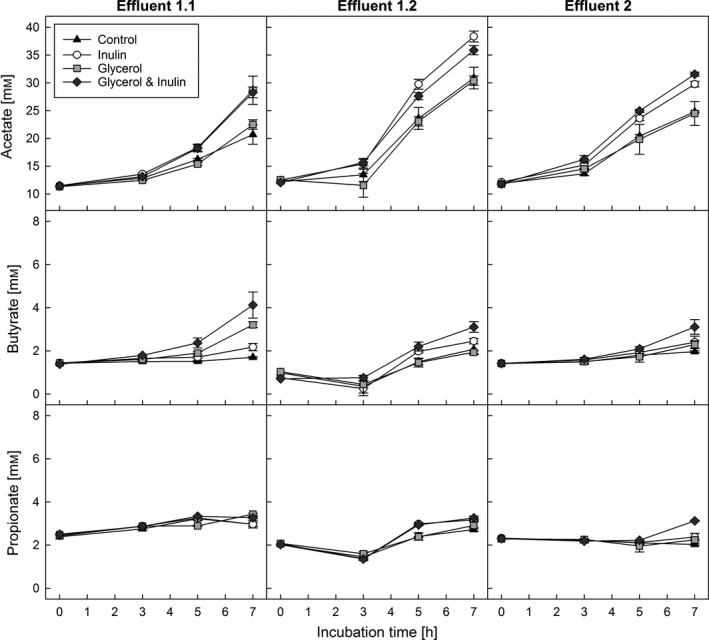
Kinetics of main metabolites production after reactivation of effluent microbiota stored for 3 months. Main metabolites acetate, propionate and butyrate were analysed by HPLC‐RI after reactivation in batch fermentations. Each point represents the average of three replicates with standard deviation.

The ability of cryoprotectants to preserve metabolic activity of effluent microbiota was determined after cryopreservation for 3 months at −80°C, using protective buffers containing inulin (5% v/w), glycerol (15% v/v) or a combination thereof. Fresh (*t*_0_) and preserved microbiota (3 months storage at −80°C), in phosphate buffer with or without (control) cryoprotectants, were used to inoculate adapted Macfarlane medium. Inoculated media were incubated for 24 h under anaerobic conditions to investigate the metabolic activity and re‐establishment of selected butyrate‐producing bacteria in batch fermentation using HPLC‐RI and qPCR respectively. Additionally, SCFA production by microbiota in preserved and reactivated effluents was determined after 3, 5 and 7 h of batch fermentation (Fig. 3). All fermentations were carried out in triplicate.

Final amounts of each SCFA produced by fresh microbiota differed among effluents but were impacted minimally by adding cryoprotectants (Table 2). SCFA amounts between the control and the treatment of microbiota 1.1 and 1.2 were similar, indicating no effect of added glycerol, inulin or combination thereof on metabolic activity. The metabolite production of microbiota 1.1 was dominated by acetate (77.9 ± 1.1–80.2 ± 4.0 mM), followed by butyrate (16.3 ± 2.7–20.7 ± 0.7 mM) and propionate (9.8 ± 3.2–13.8 ± 0.3 mM), giving a total of 107.1 ± 6.0–112.7 ± 0.87 mM SCFAs. Microbiota 1.2 produced comparable amounts of total SCFAs (107.4 ± 1.3–111.1 ± 2.2 mM), but slightly higher amounts of propionate (16.4 ± 1.4–1.9 ± 1.2 mM) at the expense of acetate (70.3 ± 1.9–74.1 ± 1.5 mM). In contrast, microbiota 2 processing with glycerol or glycerol and inulin changed final amounts of main SCFAs produced. Propionate production was significantly reduced (*P *< 0.05) compared to the control (17.7 ± 0.4 and 14.2 ± 0.2 versus 21.6 ± 0.3 mM), while butyrate production was enhanced with glycerol (16.4 ± 0.2 mM) relative to levels in the control treatment (12.9 ± 0.3 mM). After 24 h of batch fermentation, lactate and formate were not detected with fresh microbiota 1.1 and 1.2; however, formate was present with microbiota 2 (9.4 ± 1.3 mM).

**Table 2:** Production of major SCFA after 24 h batch fermentation of effluent samples prior and poststorage at −80°C

Protective buffer	Effluent 1.1			Effluent 1.2			Effluent 2		
	Prior to freezing (mM)	Postfreezing (mM)	Recovery (%)	Prior to freezing (mM)	Postfreezing (mM)	Recovery (%)	Prior to freezing (mM)	Postfreezing (mM)	Recovery (%)
Glycerol and Inulin									
Acetate	79.9 ± 0.2	70.6 ± 1.1^a^^,^ b	88.4	70.3 ± 1.9	61.7 ± 0.4^b^	87.8	54.5 ± 0.5^a^	52.7 ± 0.6^a^^,^ b	96.7
Propionate	12.2 ± 0.3	13.0 ± 0.3^a^	106.6	17.2 ± 0.6	16.8 ± 0.5	98.0	14.2 ± 0.2^a^	17.8 ± 0.2^a^^,^ b	125.3
Butyrate	17.1 ± 0.2	18.2 ± 0.7^a^	106.4	19.9 ± 1.2	19.4 ± 1.0^a^	97.5	12.9 ± 0.6	16.2 ± 0.1^b^	125.6
Total SCFA	109.1 ± 0.7	101.9 ± 0.8^a^^,^ b	93.4	107.4 ± 1.3	98.0 ± 0.3^a^^,^ b	91.1	81.6 ± 0.6^a^	86.8 ± 0.9^a^^,^ b	106.4
Glycerol									
Acetate	80.2 ± 4.0	68.6 ± 0.5^b^	85.5	70.6 ± 2.0	60.3 ± 0.5^b^	85.4	57.2 ± 1.1^a^	53.8 ± 1.3^a^^,^ b	94.1
Propionate	9.8 ± 3.2	12.7 ± 1.1^a^	129.6	16.4 ± 1.4	16.7 ± 0.9	102.1	17.7 ± 0.4^a^	15.9 ± 0.8^a^	90.0
Butyrate	17.1 ± 1.2	16.2 ± 0.6^a^	94.7	20.9 ± 1.5	17.4 ± 0.1^a^	83.5	16.4 ± 0.4^a^	16.9 ± 0.1^a^	103.3
Total SCFA	107.1 ± 6.0	97.5 ± 1.3^a^	91.0	107.9 ± 1.9	94.5 ± 1.2^b^	87.6	91.2 ± 1.0^a^	86.6 ± 1.6^a^^,^ b	95.0
Inulin									
Acetate	77.9 ± 1.1	67.6 ± 1.4^b^	86.8	71.0 ± 1.2	65.6 ± 3.1	92.4	59.4 ± 1.0	49.0 ± 0.9^b^	82.5
Propionate	13.8 ± 0.3	9.3 ± 0.4^a^^,^ b	67.4	17.7 ± 0.1	17.4 ± 1.1^a^	98.4	23.3 ± 0.3^a^	16.2 ± 0.5^a^^,^ b	69.6
Butyrate	20.7 ± 0.7	9.4 ± 0.2^b^	45.4	19.1 ± 0.5	16.0 ± 0.1^b^	83.8	14.1 ± 0.0	14.4 ± 1.2	102.0
Total SCFA	112.7 ± 0.7	86.3 ± 1.6^a^^,^ b	76.6	107.7 ± 0.9	99.0 ± 3.9^a^	91.9	96.8 ± 1.3^a^	79.6 ± 2.0^a^^,^ b	82.2
Control									
Acetate	79.7 ± 1.5	63.5 ± 3.3^b^	79.7	74.1 ± 1.5	60.5 ± 2.1^b^	81.6	59.4 ± 0.2	47.9 ± 0.2^b^	80.7
Propionate	12.6 ± 0.5	4.9 ± 0.5^b^	38.9	18.1 ± 0.5	15.4 ± 0.3^b^	85.2	21.6 ± 0.3	13.3 ± 0.4^b^	61.4
Butyrate	16.3 ± 2.7	9.9 ± 0.5^b^	60.7	18.9 ± 0.2	15.6 ± 0.8^b^	82.3	12.9 ± 0.3	13.8 ± 0.7	107.2
Total SCFA	108.6 ± 1.7	78.3 ± 3.6^b^	72.1	111.1 ± 2.2	91.5 ± 2.5^b^	82.3	93.9 ± 0.3	75.0 ± 0.9^b^	79.9

Main metabolites acetate, propionate and butyrate formed by effluents immediately after processing (*t* = 0) and after 3 months storage at −80°C were analysed by HPLC‐RI (means and standard deviations of independent fermentation triplicates).

a Indicates that metabolite formed in treatment is significantly different from the control within the same effluent microbiota (*P *< 0.05).

b Indicates that metabolite formed is significantly different prior and postfreezing within the same treatment and effluent microbiota (*P *< 0.05).

After preservation, metabolic activity of effluent microbiota was generally lower compared to fresh microbiota (Table 2). The lowest recovery of total SCFA levels compared to fresh microbiota was measured in control samples preserved without added cryoprotectants (72.1%, 82.3% and 79.9% respectively). All three main SCFAs were significantly decreased (*P *< 0.05). Glycerol alone and in combination with inulin maintained best the overall metabolic activity of the stored microbiota. The observed decrease in SCFA concentrations in microbiota samples preserved in glycerol was associated with lower acetate formation (85.5%, 85.4% and 94.1% recovery), whereas the amounts of propionate and butyrate did not significantly differ from the concentrations produced by fresh microbiota. When microbiota were preserved with glycerol combined with inulin, a decrease in acetate production was also observed (88.4%, 87.8% and 96.7% recovery). Microbiota 1.1 and 1.2 with glycerol and inulin formed similar amounts of propionate and butyrate compared to fresh microbiota, while microbiota in stored effluent 2 produced significantly higher concentrations of propionate and butyrate (*P *< 0.05; 125.3% and 125.6% respectively). Independent of the treatment, lactate and formate were detected after 7 h incubation (Fig. S1) but were not detectable anymore after 24 h batch fermentation, with the exception of cryopreserved microbiota 2, which had formate concentrations of 3.1 ± 2.1–3.9 ± 0.4 mM.

Concerning the SCFA production kinetics after storage, acetate and butyrate were detectable after 7 h incubation, whereas propionate was not detected. Microbiota 1.1, 1.2 and 2 produced more acetate in batch fermentation after 7 h incubation when preserved in inulin (28.7 ± 0.3, 38.4 ± 1.0 and 29.8 ± 0.5 mM) or glycerol and inulin (28.3 ± 0.1, 35.9 ± 0.8 and 31.6 ± 0.4 mM) compared to the control treatment (20.7 ± 1.7 mM, 30.9 ± 2.0 and 24.8 ± 0.1 mM). Glycerol in combination with inulin also increased butyrate formation (4.1 ± 0.6, 3.1 ± 0.2 and 3.1 ± 0.3 mM) compared to controls (1.7 ± 0.0, 2.1 ± 0.2 and 2.0 ± 0.0 mM) during 7 h incubation.

### Impact of cryopreservation on re‐establishment of selected butyrate‐producing bacteria

We also investigated the effect of cryoprotectants on growth and relative abundance of butyrate‐producing bacteria, *Roseburia* spp./*E*. *rectale* group, *F. prausnitzii* and *E. hallii*, during a 24 h batch fermentation using qPCR (Table 3).

**Table 3:** Growth and relative abundance of selected butyrate‐producing bacteria after 24 h batch fermentation of effluent samples prior and poststorage at −80°C

Protective buffer	Effluent 1.1				Effluent 1.2				Effluent 2			
	Prior to freezing		Postfreezing		Prior to freezing		Postfreezing		Prior to freezing		Postfreezing	
	Δlog	Rel. ab.	Δlog	Rel. ab.	Δlog	Rel. ab.	Δlog	Rel. ab.	Δlog	Rel. ab.	Δlog	Rel. ab.
Roseburia spp./*E. rectale*												
Glycerol & Inulin	1.0 ± 0.0	6.3 ± 0.6	1.0 ± 0.1^a^	6.9 ± 0.7^a^	1.0 ± 0.1^a^	4.7 ± 0.5^a^	0.1 ± 0.1^a^^,^ b	1.4 ± 0.3^a^^,^ b	1.3 ± 0.0^a^	0.2 ± 0.0	1.3 ± 0.0^a^	0.1 ± 0.0
Glycerol	0.9 ± 0.2	3.3 ± 2.6	1.0 ± 0.0^a^	6.4 ± 0.5^a^	1.2 ± 0.1	6.5 ± 0.6	−0.4 ± 0.0^a^^,^ b	0.5 ± 0.1^b^	1.4 ± 0.2^a^	0.2 ± 0.1^a^	1.7 ± 0.1^a^	0.2 ± 0.1^a^
Inulin	1.1 ± 0.1	9.9 ± 1.1	−0.2 ± 0.1^b^	0.4 ± 0.0^b^	1.2 ± 0.0	6.5 ± 0.6	−0.5 ± 0.0^a^^,^ b	0.4 ± 0.0^b^	0.7 ± 0.1	0.1 ± 0.0	0.8 ± 0.1	0.0 ± 0.0
Control	0.8 ± 0.2	4.8 ± 3.6	−0.6 ± 0.3^b^	0.2 ± 0.1	1.2 ± 0.0	7.4 ± 0.9	−0.9 ± 0.2^b^	0.2 ± 0.1^b^	0.7 ± 0.2	0.1 ± 0.0	0.8 ± 0.1	0.0 ± 0.0
*F. prausnitzii*												
Glycerol & Inulin	0.4 ± 0.0	3.6 ± 0.5	0.5 ± 0.1^a^	1.1 ± 0.1^b^	0.7 ± 0.1	0.4 ± 0.2^a^	0.6 ± 0.1^a^	1.7 ± 0.4^a^^,^ b	0.4 ± 0.1^a^	0.3 ± 0.0^a^	1.4 ± 0.1^a^^,^ b	1.2 ± 0.2^a^^,^ b
Glycerol	0.4 ± 0.1	3.8 ± 0.7	−0.4 ± 0.0^a^^,^ b	0.1 ± 0.0^b^	0.8 ± 0.0	0.4 ± 0.0^a^	0.0 ± 0.1^b^	0.5 ± 0.1	0.8 ± 0.0	0.8 ± 0.1	1.1 ± 0.0^b^	0.7 ± 0.1^b^
Inulin	0.6 ± 0.0	5.5 ± 0.2^a^	0.8 ± 0.2^a^	2.0 ± 0.3^a^^,^ b	0.5 ± 0.0^a^	0.5 ± 0.0	0.7 ± 0.0^a^^,^ b	2.5 ± 0.1^a^^,^ b	1.0 ± 0.0^a^	1.6 ± 0.1^a^	1.3 ± 0.1^a^^,^ b	1.2 ± 0.3^a^
Control	0.5 ± 0.0	4.1 ± 0.7	0.1 ± 0.0^b^	0.4 ± 0.1^b^	0.8 ± 0.1	0.5 ± 0.0	0.1 ± 0.1	0.6 ± 0.1^b^	0.8 ± 0.1	1.0 ± 0.1	0.4 ± 0.1^b^	0.1 ± 0.0^b^
*E. hallii*												
Glycerol & Inulin	2.6 ± 0.1	1.0 ± 0.1	2.5 ± 0.2	1.8 ± 0.4^a^	2.1 ± 0.0	0.5 ± 0.1	2.0 ± 0.1^a^	2.5 ± 0.3b^a^^,^ b	2.2 ± 0.0	0.6 ± 0.1	2.2 ± 0.1^a^	0.7 ± 0.1^a^
Glycerol	2.0 ± 0.4	0.3 ± 0.2	2.3 ± 0.0	1.2 ± 0.0^b^	2.0 ± 0.0	0.5 ± 0.1	1.8 ± 0.1^b^	1.8 ± 0.1	1.8 ± 0.2	0.3 ± 0.1^a^	2.2 ± 0.1^a^	0.7 ± 0.1^a^^,^ b
Inulin	2.0 ± 0.1	0.3 ± 0.0	2.3 ± 0.0^b^	1.2 ± 0.2^b^	2.1 ± 0.1	0.5 ± 0.0	1.8 ± 0.1^b^	1.7 ± 0.1^b^	2.1 ± 0.1	0.6 ± 0.1	2.0 ± 0.1	0.5 ± 0.1
Control	1.9 ± 0.0	0.2 ± 0.0	2.2 ± 0.0^b^	1.1 ± 0.1^b^	1.9 ± 0.2	0.4 ± 0.1	1.7 ± 0.1^b^	1.5 ± 0.1^b^	2.1 ± 0.1	0.6 ± 0.2	1.9 ± 0.1	0.3 ± 0.1

Growth (Δlog) was determined as log increase during 24 h batch fermentation of effluents immediately after processing (*t* = 0) and after 3 months storage at −80°C analysed by qPCR. Relative abundance (rel. ab.) was determined as ratio of target gene of respective bacterial group/total bacteria (means and standard deviations of independent fermentation triplicates).

a Indicates that growth respectively relative abundance in treatment is significantly different from the control within the same effluent microbiota (*P *< 0.05).

b Indicates that growth respectively relative abundance is significantly different prior and postfreezing within the same treatment and effluent microbiota (*P *< 0.05).

No significant difference in growth or relative abundance of targeted butyrate‐producing bacteria was observed between control and treatments containing cryoprotectants in microbiota 1.1. After 24 h of batch fermentation, average increases in 0.9 ± 0.2, 0.5 ± 0.1 and 2.1 ± 0.3 logs were observed for the *Roseburia* spp./*E. rectale* group, *F. prausnitzii*, and *E. hallii*, giving final relative abundances of 6.1 ± 3.2%, 4.2 ± 0.9% and 0.5 ± 0.3% respectively. The tested treatments had only a minor impact on growth of selected butyrate‐producing bacteria in microbiota 1.2. The *Roseburia* spp./*E. rectale* group showed significantly less growth (*P *< 0.05) and reached a lower relative abundance when treated with glycerol and inulin (1.0 ± 0.1 log and 4.7 ± 0.5% respectively) than was observed in the control (1.2 ± 0.0 log and 7.4 ± 0.9% respectively). Inulin treatment resulted in slightly decreased growth (*P *< 0.05) of *F. prausnitzii* (0.5 ± 0.0 log) compared to the control (0.8 ± 0.1 log). Growth and relative abundance of *E. hallii* in microbiota 1.2 were not affected by treatments and control (2.0 ± 0.1 log and 0.5 ± 0.1% respectively). In contrast, cryoprotective treatments strongly impacted the growth of butyrate‐producing bacteria in fresh microbiota 2. Growth of *Roseburia* spp./*E. rectale* group, which was present at low relative abundance (0.1 ± 0.0–0.2 ± 0.1%), was significantly enhanced (*P *< 0.05) by treating with protective buffers containing glycerol or glycerol and inulin (1.3 ± 0.1 and 1.4 ± 0.2 logs respectively) compared to the control (0.7 ± 0.2 log). In contrast, glycerol and inulin decreased the growth and relative abundance of *F. prausnitzii* (0.4 ± 0.1 log and 0.3 ± 0.0%) compared to the control (0.8 ± 0.1 log and 1.0 ± 0.1%). Growth of *E. hallii* (2.0 ± 0.2 logs) was not different between treatments and control.

After cryopreservation, growth of the *Roseburia* spp./*E. rectale* group in the control and microbiota 1.1 and 1.2 that had been treated with inulin was strongly impaired compared to fresh microbiota, as shown by a decrease in log gene copies after 24 h incubation (−0.9 ± 0.2 to −0.2 ± 0.1 log). In contrast, glycerol alone and in combination with inulin maintained growth and relative abundance of the *Roseburia* spp./*E. rectale* group in microbiota 1.1 to levels similar to fresh samples (1.0 ± 0.0 and 1.0 ± 0.1 log respectively). For microbiota 1.2, growth after cryopreservation was also not recovered when glycerol alone or in combination with inulin was added. In preserved microbiota 2, the *Roseburia* spp./*E. rectale* group grew after all treatments and in the control. Growth was significantly enhanced (*P *< 0.05) in the treatments containing glycerol (1.3 ± 0.0 and 1.7 ± 0.1 logs) compared to the control (0.8 ± 0.1 log), as was observed with the fresh microbiota.

Growth of *F. prausnitzii* after effluent preservation strongly depended on treatment. Control and glycerol treatment resulted in significantly impaired growth (*P *< 0.05) of *F. prausnitzii* in microbiota 1.1 and 1.2 (−0.4 ± 0.0 to 0.1 ± 0.1 log). In contrast, treatments containing inulin in microbiota 1.2 showed growth of *F. prausnitzii* (0.7 ± 0.0 log) that was equal to or greater than growth in fresh microbiota fermentation. In effluent 2, *F. prausnitzii* treated with inulin alone or in combination with glycerol (1.3 ± 0.1 and 1.4 ± 0.1 logs respectively) grew significantly better (*P *< 0.05) than in fresh microbiota. In contrast, growth of *F. prausnitzii* in the control was significantly reduced (*P *< 0.05) compared to fresh samples (0.4 ± 0.1 log).

Cryopreservation and the presence or absence of cryoprotectants had no impact on growth of *E. hallii* in microbiota 2 compared to fresh microbiota of the same condition. With microbiota 1.1, the relative abundance of *E. hallii* increased up to fivefold (1.1 ± 0.1–1.8 ± 0.4%) after incubation of preserved microbiota compared to fresh microbiota. In contrast, preserved microbiota 1.2 showed significantly decreased growth of *E. hallii* (*P *< 0.05) compared to fresh microbiota (1.7 ± 0.1–2.0 ± 0.1 logs).

## Discussion

Restoring the compositional balance of the intestinal microbiota by FMT has been proposed to treat a broad range of chronic intestinal diseases associated with microbial dysbiosis (Khoruts and Sadowsky, 2016). Artificial colonic microbiota transplants derived from intestinal fermentation technology could enhance availability, acceptability and safety, associated with faecal material. For the first time, we have described enhanced storage conditions for artificial colonic microbiota by cryopreservation with glycerol and inulin with focus on maintenance of selected butyrate‐producing bacteria, which are associated with ‘sustained remission’ of gastrointestinal diseases after FMT (Fuentes *et al*., 2017).

Metabolite analyses of effluents derived from F1 and F2 identified two distinct SCFA profiles. The microbiota exhibited butyrogenic (F1) or propiogenic (F2) characteristics. The high fraction of butyrate of F1 effluents was associated with a microbial community dominated by Firmicutes harbouring highly abundant populations of *Ruminococcaceae* and *Lachnospiraceae* including *F. prausnitzii* and the *Roseburia* spp./*E. rectale* group, respectively, next to other unknown butyrate producers. In F2, butyrate producers were also present but Bacteroidetes (mainly *Prevotellaceae*) were more abundant than Firmicutes. Bacteroidetes form propionate via succinate pathway, which is the predominant propionate pathway in adults (Reichardt *et al*., 2014). These outcomes demonstrate that IFT can reproduce, at least to a certain extent, the initial microbiota profile of the faecal donor's microbiota. Furthermore, effluent microbiota composition could be modified by short‐term pH increase. Many Firmicutes are more tolerant to low pH, whereas *Bacteroides* spp. have growth advantages at higher pH (Flint *et al*., 2012). The observed shift from Firmicutes to Bacteroidetes in F1.2 compared to F1.1 was coherent with an increase in propionate production.

As direct testing of the preserved samples with molecular methods does not provide information on the activity status of the microbiota, we used adapted Macfarlane medium to investigate growth and metabolic activity of fresh and preserved microbiota during a 24 h batch fermentation. Batch fermentations are limited by restricted substrate supply and buffer capacity (Payne *et al*., 2012). However, such fermentations allow investigation of bacterial viability and activity of the inoculum in a controlled and reproducible way. Modified Macfarlane medium contains a mix of SCFA to initiate growth of butyrate‐producing bacteria such as *F. prausnitzii* and *R. intestinalis* (Duncan *et al*., 2002a), as well as complex glycans, which provides fuel for, and can be degraded by, trophic interactions of the gut microbiota. All three investigated butyrate producers grew in batch cultures, as indicated by qPCR data. However, changes in relative abundances from effluent microbiota to batch fermentation indicate that the applied batch conditions are more favourable to *E. hallii* and the *Roseburia* spp./*E. rectale* group than to *F. prausnitzii*.

DMSO and glycerol are the most common protectants added to bacterial cells to enhance cryopreservation (Hubalek, 2003). However, low concentrations of DMSO might result in cellular toxicity (Galvao *et al*., 2014), which limits its use for FMT unless a removal step is used before transplantation. Additionally, DMSO alone showed no significant improvement in SCFA production by faecal microbiota after frozen storage and reactivation compared to the control without cryoprotectant (Kerckhof *et al*., 2014). Glycerol prevents hydrogen bonding between water molecules and thus prevents formation of intracellular ice crystals during freezing (Koh, 2013). Glycerol is commonly added before freezing and storage of faecal microbiota for FMT at −80°C (Hamilton *et al*., 2012). Here, we observed only minor losses (<10% of total SCFA production) after 24 h incubation when effluent microbiota was frozen in 15% glycerol, which is the recommended amount for freeze storage of bacterial cultures (Koh, 2013). In our study, the addition of glycerol selectively enhanced survival of members of the *Roseburia* spp./*E. rectale* group. The diverse *Roseburia* spp./*E. rectale* group, encompassing species such as *Roseburia inulinivorans* or *Roseburia intestinalis,* is estimated to represent between 5% and 10% of the faecal microbiota (Aminov *et al*., 2006; Louis and Flint, 2009). In addition to its cryoprotective action, glycerol may also serve as substrate for a broad range of microbes during reactivation (Engels *et al*., 2016). Nevertheless, the ability to grow on glycerol is not widely shared among members of the *Roseburia* spp./*E. rectale* group. Of 12 tested strains, only two strains of *Roseburia hominis* and one strain of *Roseburia ceciola* were able to utilize glycerol (Duncan *et al*., 2006). The final glycerol concentration (2 mmol l^−1^), transferred with the inoculum in batch culture, was also likely too low to induce a substrate effect. Furthermore, the positive effect of glycerol addition on SCFA formation was only observed after microbiota freezing, suggesting that glycerol acted as cryoprotectant of the *Roseburia* spp./*E. rectale* group rather than as a growth substrate in our experimental set‐up. Our results together with other studies point to glycerol as a suitable cryoprotectant for microbiota transplant cryopreservation.

*F. prausnitzii* exhibited enhanced growth in batch fermentation after freezing and storage when the cryoprotective buffer was supplemented with inulin. Inulin‐type fructans are not commonly used as cryoprotectants, despite being known as water‐soluble natural cryoprotective agents synthesized by many plant, fungi and bacteria (Hubalek, 2003). Fructans directly interact with lipids of biological membranes and stabilize them under cold as well as dry conditions (Demel *et al*., 1998; Vereyken *et al*., 2003). Inulin can also serve as a fermentation substrate, as several *F. prausnitzii* strains can use inulin (Duncan *et al*., 2002b). *In vivo*, an increased inulin intake by 10 healthy volunteers led to significantly increased numbers of *F. prausnitzii* in faecal microbiota, which indicates that *F. prausnitzii* benefits from inulin addition even in the presence of a competitive microbiota (Ramirez‐Farias *et al*., 2009). Here, the presence of inulin alone, or glycerol and inulin in combination, also decreased the lag time of acetate and butyrate formation. However, the amount of inulin supplied with the inoculum was likely too little (25 mg l^−1^) to impact final SCFA formation during fermentation. Nevertheless, inulin bound to the cell membranes during freezing could possibly act as an easily accessible nutrient source to fulfil the increased nutritional demands of stressed bacterial cells immediately after reactivation (Ray and Speck, 1973).

*E. hallii*, with a high prevalence in adults (Engels *et al*., 2016), showed the lowest initial abundance of all three targeted butyrate‐producing bacteria in effluent microbiota from both fermentation systems. Nevertheless, growth was strongly induced during batch fermentation, resulting in a more than 100‐fold increase. We reported similar increases for batch fermentation of effluents derived from IFT mimicking healthy elderly colonic microbiota (Fekry *et al*., 2016). The competitiveness of *E. hallii* in batch fermentation could be due to its ability to feed on lactate and acetate (Duncan *et al*., 2004; Belenguer *et al*., 2006). Because growth was not impacted after cryopreservation, our results also suggest that *E. hallii* is highly resistant against damage caused by freezing.

## Conclusion

This study demonstrated that combining glycerol and inulin in protective buffers provided a higher level of protection during cryopreservation of compositionally different, artificial colonic microbiota compared to single component application. Our data indicate that the functional group of butyrate‐producing bacteria, which are important for gut health but also reported to be very sensitive to environmental conditions, can be well preserved with cryoprotective agents during storage at −80°C for at least three months. The metabolic activity of frozen effluent microbiota derived from IFT and intended for FMT can be adequately maintained. The methods and preservation conditions developed in this study will be useful for further research on storage of anaerobic gut microbes and for developing new microbial‐based treatments of gastrointestinal disorders. Ultimately, preserved artificial microbiota need to be transplanted *in vivo* to test its effectiveness in a complex system involving the host and the presence of a competitive microbiota.

## Experimental procedures

### Experimental design

The freezing experiments were conducted with microbiota produced with two independent fermentation systems inoculated with immobilized faecal microbiota from different male adult donors (F1 and F2), mimicking conditions of the proximal colon. F1 effluents were used to conduct two freezing trials. The first trial was carried out with microbiota produced under standard fermentation condition (effluent 1.1) for adult proximal colon, at 37°C, pH 5.7 and a mean retention time of 8 h (Payne *et al*., 2012). Before the second trial, the microbiota composition of F1 was modulated by applying short‐term high pH stress (pH 9) to induce a lasting shift, leading to altered metabolic activity (effluent 1.2). A stabilization period at standard fermentation condition for one week was used after the pH shock before collecting effluent sample 2. With F2, one freezing trial was carried out (effluent 2). At time point 0 h of each trial, the collected microbiota material was divided into two portions, and aliquots were mixed with peptone buffer with or without protective agents. The aliquots of the first portion served as a fresh control and were used immediately to inoculate batch fermentation medium in a prior‐freezing activity test (*t*_0_). Immediately after inoculation and after 24 h of fermentation, samples were taken for DNA extraction and metabolite quantification. The aliquots of the other portion were subjected to cryopreservation for 3 months at −80°C (*t*_1_), after which reactivation in batch fermentation medium for 24 h in a postfreezing activity test was performed. An additional activity test was performed to more closely monitor the kinetics of metabolite production after 3, 5 and 7 h incubation.

### Faecal inoculum and immobilization

For each fermentation system, fresh faeces from two different adult male donors (age 30–40 years), who had not been treated with antibiotics for the last 3 months, were assigned to immobilization procedure. After defecating, approximately 5 g of faecal sample was transferred to a preweighted Falcon tube containing 5 ml of sterile, prereduced peptone water (0.1%, pH 7; Thermo Fisher Diagnostics AG, Pratteln, Switzerland) and placed in an anaerobic jar (Anaerojar, Oxoid, Hampshire, England) to maintain anaerobic condition during transport. Before immobilization in the anaerobic chamber, peptone water was added to the faecal sample to obtain a final v/w ratio of 20%. Faecal microbiota was immobilized in 1–2 mm gellan–xanthan gel beads as described previously (Zihler Berner *et al*., 2013).

### Production of complex colonic microbiota with intestinal fermentation technology

The gut microbiota used for cryopreservation tests was produced in two independent continuous colonic fermentation systems inoculated with immobilized adult gut microbiota. Sixty millilitres of freshly prepared faecal beads was transferred to a glass bioreactor (Sixfors, Ismatec, Switzerland) containing 140 ml of sterile nutritive medium, mimicking the chyme entering the colon (Macfarlane *et al*., 1998), and supplemented with a filter‐sterilized vitamin solution (Michel *et al*., 1998). In the first step, batch fermentations were carried out to colonize the beads (Fehlbaum *et al*., 2015). The bioreactors were continuously flushed with CO_2_ to maintain anaerobiosis in the fermentation system, while the temperature was kept at 37°C and the pH maintained at 5.7 by the addition of 2.5 M NaOH (Fehlbaum *et al*., 2015). Fermented medium was replaced twice by fresh medium for batch fermentations until the base consumption started to decrease. The system was set to continuous mode after the third batch fermentation by generating a constant inflow of 25 ml h^***−***1^ medium, targeting a mean retention time of 8 h. This rapid turnover is distinctive for the proximal colon region, where high supply of nutrients promotes bacterial growth leading to a dense and highly active microbiota for production of artificial gut microbiota and preservation tests (Payne *et al*., 2012). After operating in continuous mode for 10 days, microbial composition in the system was stable for collection of effluent for cryopreservation, as indicated by stable base consumption, metabolites (HPLC‐RI) and population composition (qPCR).

### Harvesting, processing and cryopreservation of microbiota

Effluent microbiota were directly collected from the bioreactors through a septum using a 120 mm needle (VWR International AG, Dietikon, Switzerland) connected to a 20 ml syringe flushed with CO_2_. Samples were transferred to a sterile prereduced 50 ml Falcon tube and transported to an anaerobic chamber (10% CO_2_, 5% H_2_ and 85% N_2_; Coy Laboratories, Grass Lake, Michigan, USA) where all further steps were performed. Effluent biomass was harvested by centrifuging 1 ml of aliquots at 10 000× *g* for 4 min. The supernatant was decanted, and the bacterial pellet was resuspended in 50 μl protective medium. The mixture was then incubated for 30 min at room temperature, taking into account the penetration time of glycerol, before snap‐freezing in liquid nitrogen and storage at −80 °C. Three microbiota samples of each protective medium were not frozen and instead immediately used for reactivation tests.

### Preparation of cryoprotective buffers

Solutions of glycerol (15% w/v; VWR International AG), inulin (5% w/v; Orafti^®^, Switzerland) and a combination thereof were prepared in phosphate‐buffered peptone water (PB) (0.1% v/w; Thermo Fisher Diagnostics AG) adjusted to pH 6.8 and supplemented with the reducing agents cysteine–HCl and riboflavin (both Sigma‐Aldrich, Buchs, Switzerland) to protect the microbiota from potential oxygen exposure during processing and storage (Khan *et al*., 2014). All components of the protective solutions were placed in the anaerobic chambers overnight to remove traces of oxygen. PB was prepared in oxygen‐free distilled water, previously boiled and bubbled with N_2_ gas. Cryoprotective agents were mixed with PB, and cysteine‐HCl and riboflavin were added to final concentrations of 1 g l^−1^ cysteine–HCl and 0.3 g l^−1^ riboflavin. PB supplemented with only cysteine and riboflavin served as control to the buffers containing cryoprotective agents. The mixtures were filter‐sterilized, wrapped in aluminium foil to protect from light and kept in the anaerobic chamber until usage.

### Preparation of batch fermentation medium

The medium used for activity tests was a nutritive medium designed to mimic the chyme entering the colon (Macfarlane *et al*., 1998), and adjusted to conditions in batch fermentation. Thus, the medium was supplemented with a SCFA mix (Duncan *et al*., 2002a), and its buffer capacity was enhanced twofold by increasing the amount of KH_2_PO_4_ and NaHCO_3_. The medium composition (g l^−1^) was as follows: 1.0 cellobiose, 1.0 xylan, 1.0 arabinogalactan, 0.5 inulin, 1.0 soluble potato starch, 3.0 casein acid hydrolysate, 5.0 bacto™ tryptone, 1.5 meat extract, 4.5 yeast extract, 4.0 mucin, 0.4 bile salt, 0.05 hemin, 0.61 MgSO_4_, 0.1 CaCl_2_∙2H_2_O, 0.2 MnCl_2_∙4H_2_O, 0.005 FeSO_4_∙7H_2_O, 0.1 ZnSO_4_∙7H_2_O, 2.0 KH_2_PO_4_, 6.0 NaHCO_3_, 4.5 NaCl and 4.5 KCl. One ml of each of Tween 80 and vitamin solution (Michel *et al*., 1998) was also added. Short‐chain fatty acids were added to supply initial nutrients (Duncan *et al*., 2002a) to give the following final concentrations: acetate (33 mM); propionate (9 mM); isobutyrate, isovalerate and valerate (1 mM each). All components of the nutritive medium were purchased from Sigma‐Aldrich, except for inulin, bile salts (Thermo Fisher Diagnostics AG), tryptone (Becton Dickinson, Allschwil, Switzerland) and KH_2_PO_4_ (VWR International AG). The pH of the medium was adjusted to 6.8 with 2.5 M NaOH. Cysteine was added to the medium to a final concentration of 1.0 g l^−1^ after boiling and gassing with CO_2_. The medium was dispensed in 20 ml portions under flowing CO_2_ into 50 ml serum flasks containing magnetic stirrers, and closed with butyl septum stoppers and aluminium caps before autoclaving.

### Reactivation in batch fermentation

Three aliquots of each protective medium were excluded from snap‐freezing and immediately underwent reactivation in a batch fermentation to serve as fresh reference in a prior‐freezing activity test (*t*_0_). Therefore, bacterial pellets mixed with either control or protective buffers were resuspended in 950 μl of batch fermentation medium to regain the initial concentration of the fermentation effluents. Each aliquot (0.2 ml) was inoculated into 20 ml of anaerobic batch fermentation medium in 50 ml serum flasks. The flasks were incubated at 37°C for 24 h under continuous stirring at 40 r.p.m. Portions (1.5 ml) of fresh (0 h) or fermented medium (24 h) were removed and centrifuged at 10 000× *g* for 5 min at 4°C. The supernatant was stored at −20°C for HPLC‐RI analysis, while the microbial pellet was stored at −80°C for DNA extraction. After storing for 3 months at −80°C, three aliquots of each protective buffer were transferred to the anaerobic chamber and thawed on ice, followed by the same reactivation procedure as described above. A similar procedure was used for testing the metabolite kinetics of preserved effluents; samples were taken for HPLC‐RI analysis after 3, 5 and 7 h of incubation.

### Quantification of butyrate‐producing bacteria

To investigate re‐establishment of the major butyrate‐producing bacteria, total genomic DNA from the stored pellets was extracted with the FastDNA SPIN kit for soil (MP Biomedicals, Illkirch Cedex, France). Butyrate‐producing bacteria were enumerated by quantitative PCR using primers targeting *butCoA* of *F. prausnitzii* or the *Roseburia *spp./*E*. *rectale* group, or the 16S rRNA gene of *E. hallii* (Table S4). The qPCR master mix contained 2x SYBR Green Mastermix (Life Technologies, Labgene Scientific Instruments, Zug, Switzerland), 0.2 μM of each forward and backward primer, and 1 μl of template genomic DNA in a total volume of 25 μl. The amplification started with a denaturation step at 95°C for 10 min, followed by 40 cycles at 95°C for 15 s and 60°C for 1 min. Melting curve analysis was performed to verify the specificity of amplification. The samples were analysed in duplicate. Standard curves were generated from 10‐fold dilution series (10^2^–10^8^ copies) of linearized plasmids containing the target genes. Relative abundance was calculated as the ratio of target gene relative to total bacterial 16S rRNA gene copies. *butCoA* gene copies were normalized to five 16S rRNA gene copies to account for several 16S rRNA gene copies in the genomes (Vital *et al*., 2013).

### Short‐chain fatty acid analysis by HPLC‐RI

The main SCFAs acetate, propionate, butyrate and two intermediate products, lactate and formate, were measured in fermentation effluents as well as in batch fermentation samples using a high‐performance liquid chromatography (Hitachi LaChrome, Merck, Dietikon, Switzerland) equipped with an Aminex HPX‐87H column (300 × 7.8 mm; Bio‐Rad, Reinach, Basel‐Land, Switzerland) and a refractive index detector as described previously (Fehlbaum *et al*., 2015). Samples were centrifuged at 13 000 × *g* for 5 min at 4°C. Undiluted supernatants were filtered through a 0.45 μm nylon membrane filter into HPLC vials and closed with crimp caps. Supernatants (40 μl injection volume) were eluted with 10 mM H_2_SO_4_ at a flow rate of 0.6 ml min^***−***1^ at 40°C. SCFAs, lactate and formate were quantified using external standards.

### Microbiota profiling with 16S rRNA sequencing

The microbial community was analysed in faecal samples and fermentation effluents. The V4 hypervariable 16S region was amplified using specific primers 515F (5′‐GTGCCAGCMGCCGCGGTAA‐3′) and 806R (5′‐GGACTACHVGGGTWTCTAAT‐3′). Sequencing was carried out on an Illumina MiSeq (StarSEQ, Mainz, Germany) using V4 chemistry for 2 × 250 bp read length. Raw sequencing reads were processed by merging the paired reads using USEARCH (v8.1.1756) with a minimum length of the merged read of 100 bp, an expected error threshold of 1 and minimum overlapping of 15 bp. Raw sequencing reads were filtered using PRINSEQ‐lite (v0.20.4) based on selected quality criteria such as: (i) no ambiguous bases; (ii) read lengths between 247 and 257 base pairs (bp); and (iii) the average quality score at 10 bp and a complexity threshold of 10. Sequences that passed quality filtering were clustered into OTUs at 97% identity level using UPARSE (usearch v8.0.1623). Representative sequences (the most abundant) for each OTU were aligned using PyNAST (QIIME‐1.8.0) and taxonomically assigned using UTAX (usearch v8.1.1756).

### Statistics

Statistical analysis of the HPLC and qPCR data (log_10_‐transformed) was performed using IBM SPSS Statistics 23.0 (IBM SPSS, Armonk, New York, USA). Concerning the HPLC data, the initial acetate and propionate concentration present in the medium were subtracted from the concentrations measured after 3, 5, 7 and 24 h batch fermentation. Data are expressed as means ± SD of three different batch fermentations inoculated with three aliquots from the same treatment or control. Data were tested for normal distribution using the Shapiro–Wilk test, and equality of variance was assessed with the Levene test. ANOVA tests were performed on every treatment to compare qPCR and HPLC data. Treatments were compared to control with a Dunnett (two‐sided) test. A nonparametric Kruskal–Wallis test was performed when data were not normally distributed or the assumption of equality of variance was violated. A paired sample *t*‐test was performed to compare data prior to freezing with postfreezing data within one treatment. Differences were considered significant for the risk α ≤ 0.05.

## Conflict of interest

The authors declare no conflict of interest.

## Supporting information


**Fig. S1.** Kinetics of intermediate metabolites production after reactivation of effluent microbiota stored for 3 months. Formate and lactate were analyzed by HPLC‐RI after reactivation in batch fermentation. Each point represents the average of three replicates with standard deviation.

**Table S1.** Relative abundance in percentage of microbial phyla in the fermentation effluents used for cryopreservation and corresponding donors.

**Table S2.** Relative abundance (in percentage) of microbial families in the fermentation effluents used for cryopreservation and corresponding donors.

**Table S3.** Concentration of major SCFAs in fermentation effluents used for cryopreservation.

**Table S4.** Primers used to quantify butyrate‐producing bacteria within the complex microbiota.
