# Supplementary figures and images for: Cryopreservation of artificial gut microbiota produced with in vitro fermentation technology
Source: Microb Biotechnol. 2017 Oct 4;11(1):163–75. doi: 10.1111/1751-7915.12844 (PMC5743790; doi:10.1111/1751-7915.12844)

Effluent 1.1

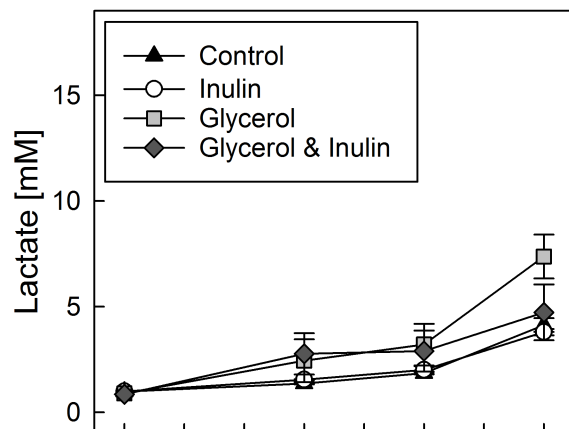

Effluent 1.2

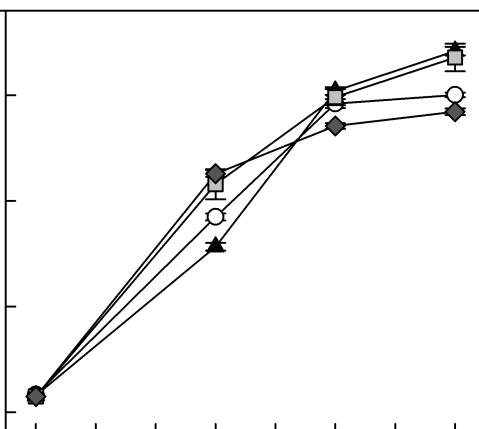

Effluent 2

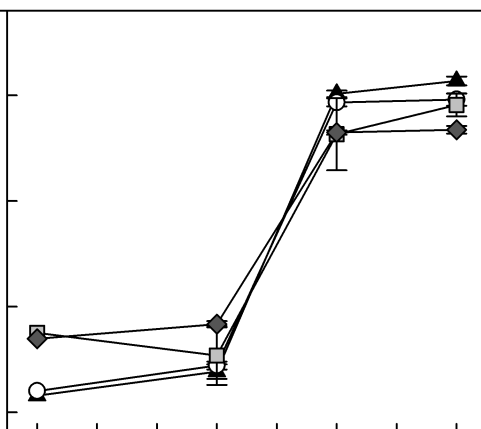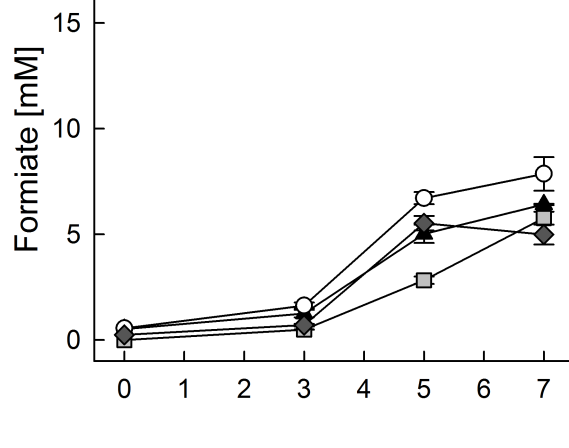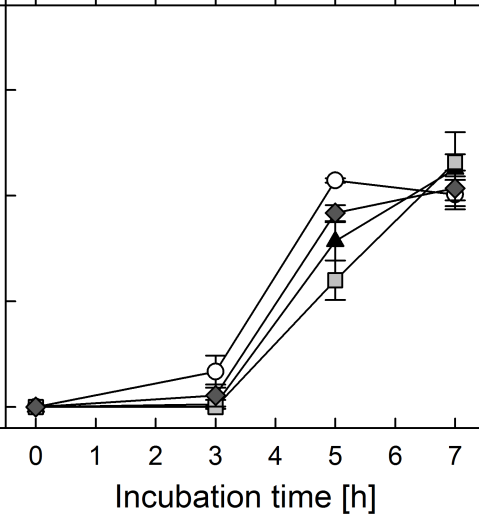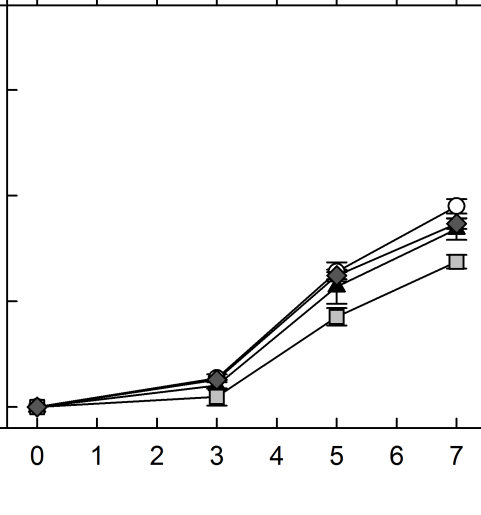

Supplement: Supplementary file 1 — Fig. S1. Kinetics of intermediate metabolites production after reactivation of effluent microbiota stored for 3 months. Formate and lactate were analyzed by HPLC‐RI after reactivation in batch fermentation. Each point represents the average of three replicates with standard deviation. [file MBT2-11-163-s001.pdf]
