# Supplementary material for: Cryopreservation of artificial gut microbiota produced with in vitro fermentation technology
Source: Microb Biotechnol. 2017 Oct 4;11(1):163–75. doi: 10.1111/1751-7915.12844 (PMC5743790; doi:10.1111/1751-7915.12844)
Supplement: Supplementary file 2 — Table S1. Relative abundance in percentage of microbial phyla in the fermentation effluents used for cryopreservation and corresponding donors. [file MBT2-11-163-s002.doc]

**Table S1.** Relative abundance in percentage of microbial phyla in the fermentation effluents used for cryopreservation and corresponding donors.

Donor 1 Effluent 1.1 Effluent 1.2 Donor 2 Effluent 2

Firmicutes 47.2 79.5 67.0 57.3 33.9

Bacteroidetes 27.9 9.1 18.5 32.7 48.7

Actinobacteria 7.2 5.2 9.7 7.2 8.6

Proteobacteria 2.5 6.2 4.8 1.1 8.8

Verrucomicrobia 5.9 0.0 0.0 0.0 0.0

Euryarchaeota 8.6 0.0 0.0 1.6 0.1

Others <1% 0.7 0.0 0.0 0.0 0.0
