# Supplementary material for: Cryopreservation of artificial gut microbiota produced with in vitro fermentation technology
Source: Microb Biotechnol. 2017 Oct 4;11(1):163–75. doi: 10.1111/1751-7915.12844 (PMC5743790; doi:10.1111/1751-7915.12844)
Supplement: Supplementary file 3 — Table S2. Relative abundance (in percentage) of microbial families in the fermentation effluents used for cryopreservation and corresponding donors. [file MBT2-11-163-s003.doc]

**Table S2.** Relative abundance (in percentage) of microbial families in the fermentation effluents used for cryopreservation and corresponding donors.

Donor 1 Effluent 1.1 Effluent 1.2 Donor 2 Effluent 2

*Enterococcaceae* 0.0 0.0 2.1 0.0 2.3

*Lactobacillaceae* 0.0 0.0 1.2 0.1 0.9

*Clostridiales* 5.8 6.8 5.3 7.5 2.9

*Clostridiaceae* 2.0 0.3 0.4 1.1 0.5

*Lachnospiraceae* 6.0 36.9 32.5 15.9 9.5

*Peptostreptococcacea* 2.2 3.5 0.3 1.3 0.0

*Ruminococcaceae* 28.4 29.5 23.2 26.2 15.3

*Veillonellaceae* 1.1 2.0 1.5 2.1 1.9

*Erysipelotrichaceae* 0.1 0.2 0.1 2.4 0.1

Other Firmicutes > 1% 1.4 0.3 0.2 0.6 0.3

*Barnesiellaceae* 1.4 0.0 0.2 0.8 0.0

*Odoribacteraceae* 1.1 0.0 0.0 1.8 0.0

*Paraprevotellaceae* 0.0 0.0 0.0 2.0 4.9

*Bacteroidaceae* 15.6 7.5 17.6 8.4 1.8

*Porphyromonadaceae* 1.6 1.5 0.5 1.7 0.2

*Prevotellaceae* 0.1 0.0 0.1 14.6 41.7

*Rikenellaceae* 5.9 0.0 0.1 3.4 0.0

S24-7 2.2 0.0 0.0 0.0 0.0

*Bifidobacteriaceae* 3.8 2.8 7.1 4.6 8.1

*Coriobacteriaceae* 3.4 2.4 2.5 2.6 0.4

*Alcaligenaceae* 0.3 6.1 4.0 0.8 1.1

*Desulfovibrionaceae* 1.9 0.0 0.0 0.1 2.1

*Enterobacteriaceae* 0.2 0.0 0.0 0.1 3.1

*Xanthomonadaceae* 0.0 0.0 0.0 0.0 2.4

*Verrucomicrobiaceae* 5.9 0.0 0.0 0.0 0.0

*Methanobacteriaceae* 8.6 0.0 0.0 1.6 0.1

Others >1% 1.0 0.1 0.8 0.2 0.1
