# Supplementary material for: Cryopreservation of artificial gut microbiota produced with in vitro fermentation technology
Source: Microb Biotechnol. 2017 Oct 4;11(1):163–75. doi: 10.1111/1751-7915.12844 (PMC5743790; doi:10.1111/1751-7915.12844)
Supplement: Supplementary file 4 — Table S3. Concentration of major SCFAs in fermentation effluents used for cryopreservation. [file MBT2-11-163-s004.doc]

**Table S3.** Concentration of major SCFAs in fermentation effluents used for cryopreservation.

Metabolite concentration [mM]^a^

Effluent 1.1 Effluent 1.2 Effluent 2

Acetate 62.3 43.1 71.1

Propionate 12.5 19.0 36.6

Butyrate 46.8 56.6 29.3

Total SCFA 121.6 118.7 136.9

**a.** Metabolite concentration of main SCFA from single measurement
