# Supplementary material for: Cryopreservation of artificial gut microbiota produced with in vitro fermentation technology
Source: Microb Biotechnol. 2017 Oct 4;11(1):163–75. doi: 10.1111/1751-7915.12844 (PMC5743790; doi:10.1111/1751-7915.12844)
Supplement: Supplementary file 5 — Table S4. Primers used to quantify butyrate‐producing bacteria within the complex microbiota. [file MBT2-11-163-s005.doc]

**Table S4.** Primers used to quantify butyrate-producing bacteria within the complex microbiota

Primers Sequence Target gene Reference

G_Fprsn_F gacaagggccgtcaggtcta *Faecalibacterium praunitzii but* Vital *et al.* (2013)

**G_Fprsn_R** ggacaggcagatRaagctcttgc

**G_RosEub_F** tcaaatcMggIgactgggtWga *Roseburia* spp./*E.rectale* group *but* Vital *et al.* (2013)

**G_Ros_R** tcgataccggacatatgccaKgag

**G_Eub_R** tcataaccgcccatatgccatgag

EhalF gcgtaggtggcagtgcaa *Eubacterium hallii* 16S rRNA gene Ramirez-Farias *et al.* (2008)

**EhalR** gcaccgragcctatacgg

Eub 338F actcctacgggaggcag Total Bacteria 16S rRNA Guo *et al.* (2008)

Eub 518R attaccgcggctgctgg
